# Stochastic amplitude-modulated stretching of rabbit *flexor digitorum profundus* tendons reduces stiffness compared to cyclic loading but does not affect tenocyte metabolism

**DOI:** 10.1186/1471-2474-13-222

**Published:** 2012-11-14

**Authors:** Thomas H Steiner, Alexander Bürki, Stephen J Ferguson, Benjamin Gantenbein-Ritter

**Affiliations:** 1 Institute for Surgical Technology and Biomechanics, University of Bern, Stauffacherstrasse 78, Bern, CH-3014, Switzerland; 2 Institute for Biomechanics, ETH Zürich, Zürich, Switzerland

**Keywords:** Tendon, Tensile stiffness, Stochastic amplitude-modulation, Strain control, Proteoglycan production, Gene expression, Cell activity

## Abstract

**Background:**

It has been demonstrated that frequency modulation of loading influences cellular response and metabolism in 3D tissues such as cartilage, bone and intervertebral disc. However, the mechano-sensitivity of cells in linear tissues such as tendons or ligaments might be more sensitive to changes in strain amplitude than frequency. Here, we hypothesized that tenocytes *in situ* are mechano-responsive to random amplitude modulation of strain.

**Methods:**

We compared stochastic amplitude-modulated versus sinusoidal cyclic stretching. Rabbit tendon were kept in tissue-culture medium for twelve days and were loaded for 1h/day for six of the total twelve culture days. The tendons were randomly subjected to one of three different loading regimes: i) stochastic (2 – 7% random strain amplitudes), ii) cyclic_RMS (2–4.42% strain) and iii) cyclic_high (2 - 7% strain), all at 1 Hz and for 3,600 cycles, and one unloaded control.

**Results:**

At the end of the culture period, the stiffness of the “stochastic” group was significantly lower than that of the cyclic_RMS and cyclic_high groups (both, p < 0.0001). Gene expression of eleven anabolic, catabolic and inflammatory genes revealed no significant differences between the loading groups.

**Conclusions:**

We conclude that, despite an equivalent metabolic response, stochastically stretched tendons suffer most likely from increased mechanical microdamage, relative to cyclically loaded ones, which is relevant for tendon regeneration therapies in clinical practice.

## Background

Tendinopathy is the term used to describe the pathological conditions resulting from tendon overuse [1,2]. The morbidity of tendon injuries, especially in sports and in manual occupations, is relatively high in our society [3,4]. Chronic tendon injuries are often associated with forceful or repetitive loading, which leads to the accumulation of micro-tears [2,5]. The relationship between repetitive mechanical loading and tenocyte metabolism has been previously investigated in several *in vitro* studies to investigate the influence of frequency, amplitude and time on the biochemical and biological response [6]. Recently, the biomechanical response of tenocytes was modeled under a variety of physiologically relevant frequency-modulated loading regimes [7-9]. Several studies demonstrate the regulation of MMP through the interaction of mechanical loading [6,10,11].

Thus, the mechano-biological response for linearly-oriented, viscoelastic tissues loaded with frequency modulation has been relatively well studied. However, from a patient’s perspective, stochastic loading may be a much more relevant scenario, since it mimics the random, physiological motions experienced in daily activities. Previous applied loading regimes found in the literature are based on a regular cyclic loading applied at different frequencies with different magnitudes [6,10-13]. Smooth and regular amplitudes do not reflect the situation *in vivo*. This has been demonstrated in *in vivo* gate analysis in rabbit, a common model selected for tendon studies, which revealed that the frequency in “relaxed” hopping is approximately 1Hz but [14] variable. Another study used the rabbit *flexor digitorum profundus* model for flexor tendon tissue engineering, where the authors found bioreactor cyclic strain increases construct strength [15]. Thus, this rabbit tendon has been successfully evaluated for a model system for the study of tendon mechano-biology multiple times in the literature [5,14].

**Figure 1:**
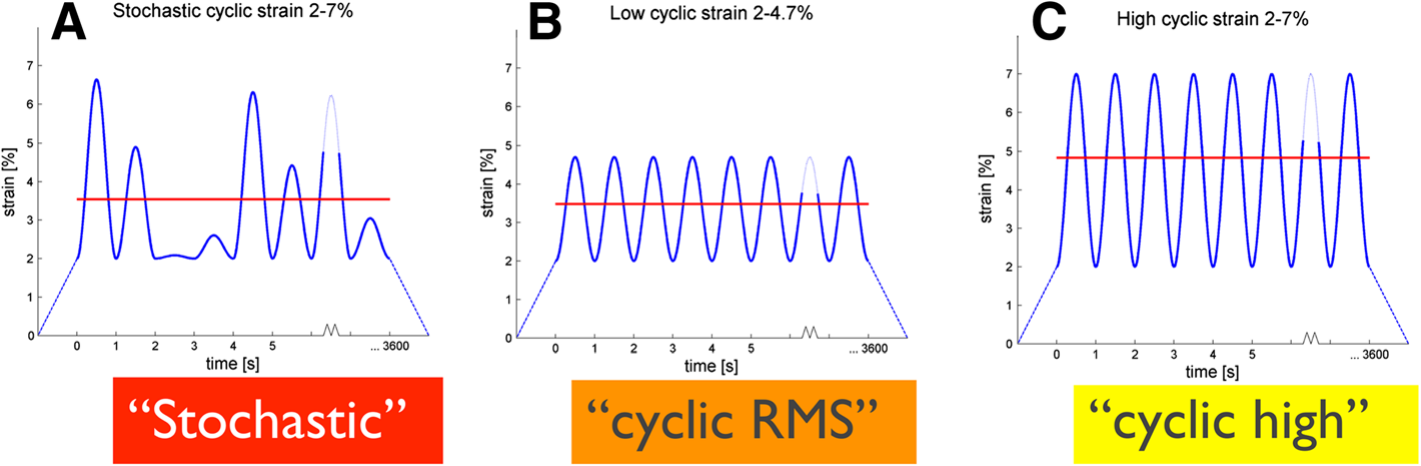
**The three different amplitude-modulated sinusoidal loading waves, which were applied in the experiment.** (**A** and **B**, both with equal root mean square [RMS] values = red lines). All regimes were run for 1 h at F = 1Hz. **C** with a higher RMS value A: low cyclic regime with the same RMS-value as the stochastic loading pattern (B). Regime C is a cyclic loading between 2-7% strain but has a higher RMS than A and B.

The aim of this study was to compare the cellular, mechanical and viscoelastic responses of tendons subjected to either a stochastic cyclic stretching or a sinusoidal cyclic stretching regime, under controlled *in vitro* conditions (see Figure 1). We hypothesize that a stochastic loading regime, applied to freshly isolated rabbit *flexor digitorum profundus* tendon, will invoke a different biochemical and biomechanical response than a symmetric, sinusoidal loading regime with an equivalent root mean square (RMS) amplitude. Furthermore, we hypothesize that a loading regime with a higher, potentially non-physiological RMS amplitude, would then shift the balance to a catabolic response of the tenocytes.

## Methods

### Tendon source and tissue harvest

Two hind paws of eight six-month old female rabbits (*Oryctolagus cuniculus*) were obtained from a local butcher within 24 h post mortem. First the hair of the hind paws was shaved and then the skin was aseptically cut and removed. After a general surface disinfection step with 1% betadine B solution (Mundipharma, Basel, Switzerland), the flexor digitorum profundus tendons (6 tendons per animal) were aseptically isolated by dissecting the muscles and immediately placed in high-glucose Dulbecco’s Modified Eagle Medium (DMEM, Gibco, Invitrogen, Basel, Switzerland) with 10% penicillin/streptomycin (1 mg/mL, Sigma) for 30 min at 37°C. Then, the specimens were washed with phosphate buffered saline (PBS) and randomly assigned to the three specified loading regimes and an unloaded control group, which was maintained in static culture conditions. The tendons were then cultured in high-glucose DMEM containing 5 μg/mL amphotericin B (Sigma) and 100 μg/mL penicillin/streptomycin containing 10% Fetal Calf Serum (FCS) at 37°C, 5% CO_2_ and 100% humidity. Media changes were performed every two days.

### Tendon stretching protocols

According to Wang et al.[4] and Wren et al. [16] and some initial pilot tensile testing, the minimal and maximal strain values defining a physiological range were set to 2 and 7%, respectively, to remain within the linear region of the load–displacement curve. Three test groups were defined, according to the loading regime applied to stretch the tendons: “stochastic”, “cyclic_RMS” and “cyclic_high” (Figure 1). The stochastic regime (”stochastic”) comprised 3,600 random stretch amplitudes between 2-7% strain. For the second group (“cyclic_RMS”), a regular sinusoidal loading regime was defined, whereby the RMS amplitude of stretching was matched to that of the stochastic loading regime. The root mean square (RMS) was calculated using the following equation:

$$f_{\mathrm{RMS}}=\lim_{Tn\to \infty }\sqrt{\frac{1}{2T}\overset{T}{\underset{-T}{\int }}\left[f{\left(t\right)}^{2}\right]dt}\;$$

This resulted in a loading regime comprising 3600 stretching cycles between 2–4.4% strain. The third group (“cyclic_high”) provided a comparison to a loading regime comprising sinusoidal stretching between the same maximal strain peaks of 2-7% strain included in the stochastic loading regime.

**Figure 2:**
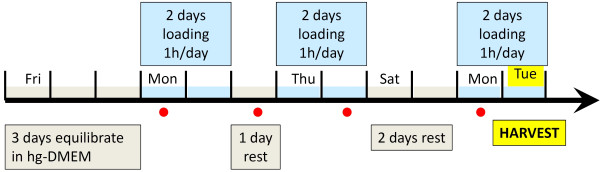
**The experimental design of the strain-controlled loading.** Upon dissection, the tendons are fixed into the loading device and allowed to equilibrate in the high-glucose DMEM cell culture medium for three days. Then, the specimens are loaded for one hour each day for two days followed by a resting day and another two days of testing. After a two-day rest another two days of testing are performed (a total of 6 days of loading, i.e. 6 hours) before the tendons are harvested and prepared for analysis. Red circle = timepoints for media changes.

**Figure 3:**
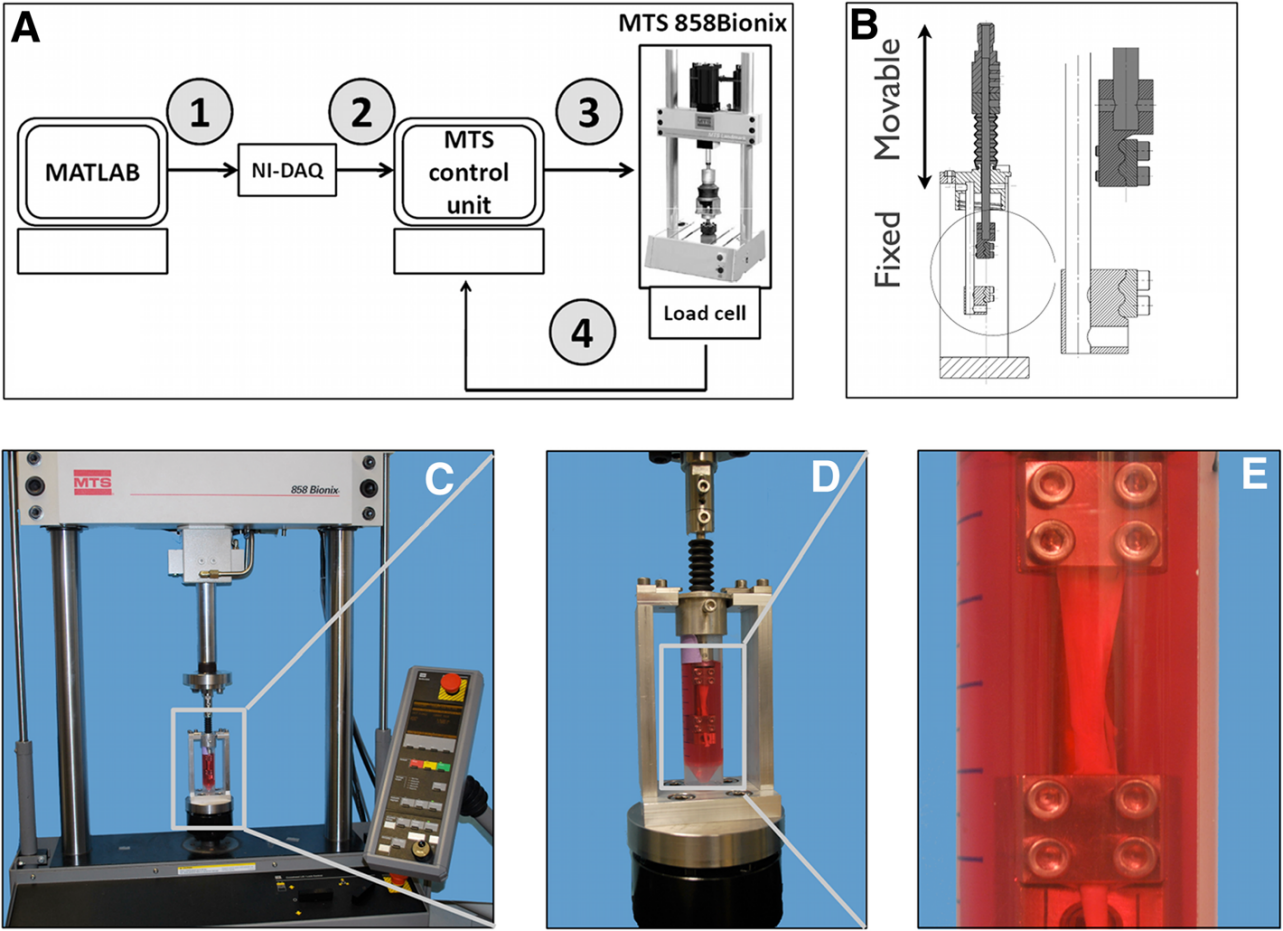
**Experimental set-up of strain-controlled mechanical loading on mechanical testing machine (MTS) A.** Conversion from digital to analog signals, 1: MATLAB generated signal (stochastic, high or RMS-cyclic signal is sent to NI-DAQ as a digital signal. 2: NI-DAQ converts digital to analog output. 3: The analog signal is scaled by the MTS control unit. Depending on the initial length of the specimen, the displacement is calculated in percentage strain. While the specimen is loaded, the control unit records the force reaction measured by the load cell (4). **B**. Construction detail of clamp. **C-E**. Set-up of strain control and force response measurement with live tendons. RMS = Root Mean Square.

Loading was applied to each tendon specimen according to the schedule in Figure 2. Loading was performed on an MTS Bionix 858 (MTS Systems, Eden Prairie, Minnesota, USA). Figure 3 shows the device mounted on the testing machine. Initial grip-to-grip length was standardized to 20 mm. The frequency was kept constant at 1Hz for all loading regimes. When the tendons were not dynamically loaded, they were constantly loaded with a pre-strain of ~1%. The tendons were stored at 37°C under standard conditions (see above). The mechanical loading was performed at room temperature, however, since the loading was applied only 1h, the cooling is similar to a media change event.

A pre-load of 2.5 N was applied to define a consistent zero strain point. The recorded output parameters were time, displacement and force response of the specimen under strain control. The data were analyzed using a custom analysis script in Matlab (Mathworks inc., MA, USA). For each of the 3,600 loading cycles, the stiffness was calculated by a linear regression of the linear portion of the loading curve. To exclude background noise from the load cell, the data was filtered and cycles with forces < 3N were omitted. Tendons that ruptured during the experiments were re-clamped (thus, shortened) and the same loading protocol was applied.

### Biochemical assays

A predefined mid-section of the tendon was used for biochemical analysis. Half of the tissue was used to assess gene expression and the other half served for the measurement of cell viability, matrix production and the DNA/GAG assay. A day 0 control was taken after the unloaded equilibration phase and processed similarly.

### RNA Extraction and Real-Time RT-PCR

The tendons were snap frozen in liquid nitrogen and pulverized with a mortar and pistil. The minced tissue of the specimens was either placed in 1ml TRI reagent (Molecular Research Center, Cincinnati, USA) and stored at −80°C prior to further RNA isolation, or was processed immediately for DNA and GAG quantification, respectively. A combined TRI phase separation-silicon column purification [17] RNA isolation was then performed with the total mammalian RNA extraction kit RTN70 (Sigma, Buchs, Switzerland). The total RNA was treated with DNaseI (DNase I amplification grade, Sigma) before the cDNA was synthesized (iScript cDNA synthesis Kit, BioRad, Basel, Switzerland). Relative gene expression was determined using the primers listed in Table 1. Along with five anabolic genes, also four catabolic and two inflammatory genes were investigated and the C_t_ threshold values were recorded. The primers and cycling protocols have been recorded previously by our group [18]. The C_t_ values were interpreted according to the 2^−∆∆Ct^-method [19].

**Table 1:** Primers used for RT-PCR

	**Abbreviation**	**Gene description**	**forward primer**	**reverse primer**
Reference gene	18S	**Reference gene 18S ribosomal RNA**	AGT GCG GGT CAT AAG CTT GC	GGT GTG TAC AAA GGG CAG GG
	ACAN	**Aggrecan**	GAG GTC GTG GTG AAA GGT GT	GTG TGG ATG GGG TAC CTG AC
Anabolic	Col1	**Collagen type 1**	TTC TTG GTG CTC CTG GCA TTC	GCA ATC CGT TGT GTC CCT TTA TG
	Col2	**Collagen type 2**	GAC CCC ATG CAG TAC ATG CG	CCA GTA GTC ACC GCT CTT CC
	MMP-1	**Metalloproteinase-1**	ATA CCT GGA AAA CTA CTA CA ATC TG	TCT TCA GGG TT TCA GCA TCT
Catabolic	MMP-3	**Metalloproteinase-3**	AGC CAA TGG AAA TGA AAA CTC TTC	CCA GTG GAT AGG CTG AGC AAA
	MMP-13	**Metalloproteinase-13**	TGC CCC TCC TCA ACA GTA AC	GAG CCC GCT GCA TTC TTC TT
	TIMP1	**Tissue inhibitor of metalloproteinase 1**	AGC AGA GCC TGC ACC TGT GT	CCA CAA ACT TGG CCC TGA TG
	TIMP3	**Tissue inhibitor of metalloproteinase 3**	TCT GCA ACT CCG ACA TCG TG	CGG ATG CAG GCG TAG TGT T
	ADAMTS4	**A disintegrin and metalloproteinase with thrombospondin motifs 4**	GAC CTT CCG TGA AGA GCA GTG T	CCT GGC AGG TGA GTT TGC AT
	TNFα	**Tumor necrosis factor alpha**	CAG CCT CTT CTC TTT CCT GCT	CCG ATC ACC CTG AAG TGC
Inflammatory	IL1b	**Interleukin-1beta**	TCC AGA CGA GGG CAT CCA	CTG CCG GAA GCT CTT GTT G
	IL-6	**Interleukin-6**	CTG GTG GTG GCT ACC GCT TT	ATG GTC TCC AGG ATG CTC CG
	IL-8	**Interleukin-8**	CAA CCT TCC TGC TGT CTC TG	GGT CCA CTC TCA ATC ACT CT

### DNA and GAG Quantification

The tendon samples were digested in 1 mL proteinase K solution for 16 h at 56°C and 300 rpm to assess both the DNA and the GAG content of the tendon. For DNA analysis, samples were stained with Hoechst dye and the fluorescent emission was measured at 457 nm with an excitation wavelength of 368 nm (Tecan Reader Infinite 200; Tecan, Männedorf, Switzerland). To measure the GAG content, the 1,9-dimethylmethylene blue (DMMB) assay, adjusted for low pH, was performed as described in Enobakhare [20] and Farndale [21] and absorbance was read at 600 nm (SpectraMax 190, Molecular Devices, Sunnyvale, California, United States, distributed by Bucher inc., Switzerland). Since the DNA content is constant per cell (~7 pg), this parameter was used to normalize both matrix production and cell activity.

### Alamar blue© cell activity test

To assess tenocyte viability after the 12-day tissue culture period, an Alamar Blue© test (Invitrogen) was performed, where the tissue was allowed to react for 2 h at 37°C and the absorbance at 570nm was measured using an absorbance reader (Tecan).

### Statistical analyses

Stiffness was analyzed using two-way ANOVA, with culture time and loading regime as the two independent factors. The gene expression, GAG/DNA and the Alamar Blue data were analyzed with non-parametric Kruskal-Wallis test using GraphPad Prism v. 6.0a, GraphPad Software, San Diego California USA, http://http//:www.graphpad.com. A p-value < 0.05 was considered significant.

## Results

**Figure 4:**
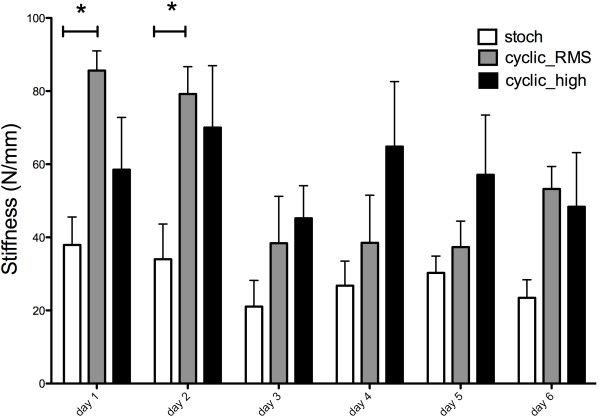
**Mean Stiffness of Tendons over time after 6 days of repetitive loading.** *Bonferroni post-hoc P < 0.05.

Mean stiffness differed (Figure 4) significantly between groups and was dependent on both factors loading and culture time (2-way ANOVA, loading explained 15.08% of variance, P < 0.0001 and time explained 9.08% of the total variance, P = 0.0116, with rabbits as a random factor). There were significant differences between time points in the cyclic_RMS group using multiple pairwise comparison testing, i.e. between day 1 vs. day 3 and day 1 vs. day 4, day 1 vs. day 5 and between day 2 vs. day 3, day 2 vs. day 4 and between day 2 vs. day 5, no such differences were found in the other two groups.

Generally, proliferation and cell activity, i.e. DNA content and Alamar blue assay, both confirmed that the tenocytes were metabolically active and alive. The "cyclic_high" group showed a slight decrease of DNA content, whereas the tendons in the other groups showed similar cell activity, but no significant difference could be found. The different tendons showed a high variance, not only the specimens from different animals, but also tendons from the same rabbit.

**Figure 5:**
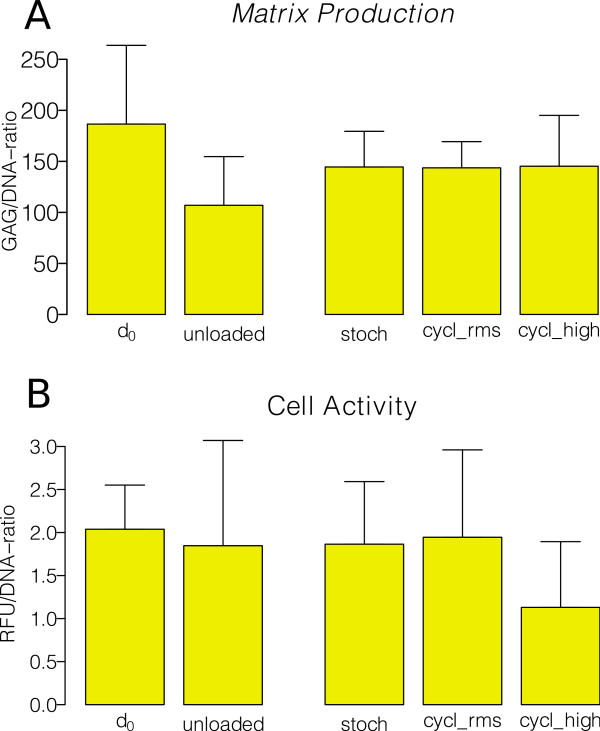
**Glycosaminoglycan (GAG) Production and Cell Activity A GAG/DNA content B Cell activity normalized to the DNA content.** The activity was different for all the loading groups and the control groups. For the loading group loaded with a high cyclic regime the relative cell activity is lowest, however, not statistically significant. For d_0_ and unloaded control: n=4; loading groups: n = 8. All values indicated as mean ± SD.

Matrix production, expressed as the glycosaminoglycan GAG/DNA content ratio, was not significantly different between the groups after culture (Figure 5A).

Cell activity (Figure 5B), as measured from Alamar blue assay, was not significantly different between all groups.

**Figure 6:**
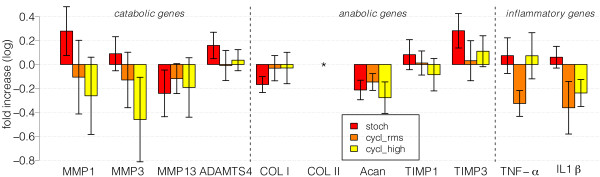
**Relative gene expression with the unloaded control as reference.** The x- fold increases are plotted on a linear scale with the average log10 values. Left: catabolic genes, middle anabolic genes, right: the inflammatory genes. Statistical analysis revealed no significant difference between the groups. For d0 and unloaded control: n = 4; loading groups: n = 8. All values indicated as log10 fold increase ± SEM. *: Collagen II was not detectable.

Relative gene expression of major catabolic and anabolic genes, relative to unloaded controls on the same culture day, revealed that “stochastic” loading tended to up-regulate metalloproteinases (i.e. MMP1 and MMP-3, ADAMTS-4, Figure 6) but also pro-inflammatory genes, such as TNF-α and IL-1β, compared to "cyclic_RMS" and "cyclic_high" loading. Collagen type 1 remained unchanged and collagen type 2 was not detectable (Figure 5). ACAN (aggrecan) was down-regulated in all groups. A parallel increase of expression of MMP-inhibitors such as TIMP-3 in the “stochastic” group weakened the up-regulation of the MMP1,-3- and 13.

## Discussion

### Mechanical properties of randomly amplitude-modulated tendons

The primary goal of this study was to test the importance of amplitude modulation for the mechanical stimulation of linearly-oriented tissues. We found significant differences in tensile stiffness between the stochastically loaded and the cyclic, sinusoidal loaded tendons (with equivalent RMS amplitude) in the first two days of loading (Figure 4). Generally, the stiffness of tendons of the stochastically stretched group was reduced, compared to the cyclically loaded tendons (Figure 4). We cannot explain this difference in stiffness strictly by biological changes, such as cell viability or activity of tenocytes (Figures 5 and 6), since we did not see any significant changes in cell viability, activity or matrix production. Furthermore, it is unlikely that metabolic changes would immediately result in observable matrix degradation. Thus, the differences are probably purely mechanical, by microfracture of collagen fibers. This should be investigated in future experiments using histological analysis at the μm scale or by SEM.

### Biological response of tenocytes

Relative to the day 0 control, all three groups of tenocytes responded with a minor down-regulation of ACAN and collagen type 1 (Figure 6). However, tenocytes of the stochastic loading regime tended to down-regulate ACAN, collagen type I, ADAMTS4 and MMP13 relative to the cyclic_RMS and cyclic_high group. An increase in collagen I with cyclic loading was also found by Wang *et al.*[4] and Parkinson 2010 *et al*. [22] observed that there is a net proteoglycan content increase in injured tendons, due to an altered metabolism rather than due to changes in gene expression levels. However, there was no difference between the loading groups and the unloaded control in the present experiments, which is also true for the up-regulation of other genes. It should be mentioned that the measured gene expression is possibly a mixture of tenocytes and progenitor cells due to the relatively young age of the rabbits.

Culture time was certainly a limit of the study; it is possible that changes to the extracellular matrix (ECM) cannot be seen with a culture period of only twelve days. On the one hand, any changes in gene expression should be still detectable, since RNA changes can be found within hours upon mechanical loading [23]. The timing of the culture start (here allowing an equilibration period of 3 days) will most likely have a detrimental influence on the mRNA transcript level, not so for col 1, but definitely for MMP3 and MMP13; these transcript levels have been shown to increase over time in an explant model of rat tail tendon fascicles [24]. With respect to tissue homeostasis, we did not find any significant differences among the three loading regimes. On the other hand, it may also be that the sampling window for gene expression was delayed and thus, no changes in RNA could be detected after the stimuli. However, it has been reported that changes in mRNA persist after 24h incubation time [23,25]. The time point of harvest after the loading regime still seems to be critical, there have been significant changes found if tissue is analyzed after 1h or longer time periods

An up-regulation of the pro-inflammatory genes that could lead to apoptosis was not evident at the RNA level in our study. Further investigation by histology or scanning electron microscopy would allow inferring definitive conclusions on the microstructure of the tendons. During the first 1–2 days of mechanical loading, 8 out of 40 tendons experienced partial ruptures and had to be re-clamped (3 in the stochastic; 2 in the cyclic_RMS and 3 in the cyclic_high group). Thus, the re-clamping of the tendons might have had an influence on the stiffness results, but may also indicate the accumulation of micro-damage. Improved clamping techniques may allow a more unbiased comparison of the clamped versus unclamped regions [26].

The cell density of the tendon is relatively low compared to other musculoskeletal tissues. This also includes the vascular cells and synovial cells of the tendon sheath that encloses each tendon [3]. The tissue is sparsely vascularized and the main constituent is collagen type 1 [27] Collagen is the main component of most organic matrices like bones, ligaments, tendons and the intervertebral disc [16]. A remarkable 60-85% of a tendons dry weight is assigned to type I collagen. A small, mechanically important portion (2%) is elastin and 4-5% are different proteins. The extracellular substance is dominated by proteoglycans (PG) and, in combination with water, they are thought to have a spacing and lubricating role for tendon [27-29]. The mechano-biological response might be masked by the generally very rich culture media, which has an abundance of growth factors, high glucose content and vitamins. Results from the matrix production at the protein level should also be reflected by the gene expression data. For all 11 genes studied, there were no statistical and biologically significant changes amongst the loading groups. These results are consistent with studies in human achilles tendon, where no changes in the expression for genes of the major collagens and proteoglycans could be found [22]. The same study also did not see any change for ADAMTS-4, MMP3, MMP13 and TIMP3 with the exception of the up-regulation of TIMP1. The authors hypothesize that the matrix turnover is favored for degeneration rather than matrix generation. However, another limitation of this study is that we did not look at tenocyte specific transcription factors such as scleraxis, which have been shown to respond to mechanical stimulation, especially with increased cyclic compression [30-32] nor did we look at tenomodulin and tenascin-C [33], two marker genes, which are important for maintaining tenocyte phenotype [34,35]. For MMP1 and MMP3, it was found that cyclic mechanical loading inhibits their expression [6,36]. It is generally accepted that training promotes both synthesis and degeneration and the process is highly dynamic [4]. It is important to state that by analysis of only RNA expression-levels, conclusions on protein expression are limited. Translation efficiency, post-translational modification and -activation, protein turnover rates or inhibitory proteins that may have a large influence on how much protein is actually synthesized. MMP could be present in the tissue as pro-MMP, and thus in an inactive form, or they might be bound to TIMPs. An up-regulation of a MMP does therefore not necessarily mean matrix degeneration [2]. Due to these potential effects it would be crucial to also include quantification on the protein level to support real-time PCR data if longer loading / culture times will be chosen in further experiments.

## Conclusions

Stochastic modulation of amplitude in strain-controlled stretching of tendons resulted in a reduced tendon stiffness, compared to sinusoidal, cyclic loading regimes, with equivalent RMS amplitude, or sinusoidal, cyclic loading between the same peak strain magnitudes. The change in stiffness was not associated with changes in cell activity, cell density (DNA) or GAG content.

## Competing interest

The author(s) declare that they have no competing interests.

## Authors’ contributions

TS: data collection, preparation of manuscript, experimental design and interpretation; AB: experimental design and mechanical testing protocols; SJF and BG: experimental design, guidance and funding. All authors read and approved the final manuscript.

## Pre-publication history

The pre-publication history for this paper can be accessed here:

http://www.biomedcentral.com/1471-2474/13/222/prepub
